# Standard psychological consultations and follow up for women at increased risk of hereditary breast cancer considering prophylactic mastectomy

**DOI:** 10.1186/1897-4287-7-6

**Published:** 2009-03-31

**Authors:** Murly BM Tan, Eveline MA Bleiker, Marian BE Menke-Pluymers, Arthur R Van Gool, Silvia van Dooren, Bert N Van Geel, Madeleine MA Tilanus-Linthorst, Karina CM Bartels, Jan GM Klijn, Cecile TM Brekelmans, Caroline Seynaeve

**Affiliations:** 1 Department of Psychosocial Care, Family Cancer Clinic, Erasmus MC-Daniel den Hoed Cancer Center, Rotterdam, The Netherlands; 2 Department of Psychosocial Research and Epidemiology, Netherlands Cancer Institute, Amsterdam, The Netherlands; 3 Department of Surgical Oncology, Family Cancer Clinic, Erasmus MC-Daniel den Hoed Cancer Center, Rotterdam, The Netherlands; 4 Department of Medical Psychology and Psychotherapy, Erasmus MC, Rotterdam, The Netherlands; 5 Department of Medical Oncology, Family Cancer Clinic, Erasmus MC-Daniel den Hoed Cancer Center, Rotterdam, The Netherlands; 6 The Central Committee on Research Involving Human Subjects, The Hague, The Netherlands

## Abstract

**Background:**

Women at increased (genetic) risk of breast cancer have to weigh the personal pros and cons of prophylactic mastectomy (PM) as an option to reduce their cancer risk. So far, no routine referral to a psychologist has been investigated for women considering PM. Aim of this study was to asses: 1) the acceptance of the offer of a standard psychological consultation as part of pre-surgical decision-making in high-risk women, 2) reasons for PM and reasons for postponing it, 3) the need for additional psychological interventions, and factors associated, and 4) the frequency of psychiatric/psychological treatment history.

**Methods:**

During a 30 months period, women at high risk considering PM were offered a psychological consultation. The content of these, and follow-up, consultations were analyzed.

**Results:**

Most women (70 out of 73) accepted the psychological consultation, and 81% proceeded with PM. Main reasons for undergoing PM were to reduce anxiety about cancer, and to reduce the cancer risk. Uncertainty about surgery and the need for further information were the reasons given most frequently for postponing PM. Additional psychological support was given to 31% before and 14% after PM. The uptake of additional support was significantly higher in women with a BRCA1/2 mutation. A history of psychiatric/psychological treatment was present in 36%, mainly consisting of depression and grief after death of a mother.

**Conclusion:**

The uptake-rate of the standard psychological consultation indicates a high level of acceptability of this service for women deciding about PM. Since anxiety is one of the main reasons for considering PM, and depression and grief were present in a third, a standard consultation with a psychologist for high-risk women considering PM may be indicated. This may help them arrive at an informed decision, to detect and manage psychological distress, and to plan psychological support services.

## Background

Prophylactic mastectomy (PM) has become a treatment option for those women identified as being at high risk for developing breast cancer. Studies on PM consistently show a risk reduction of breast cancer of at least 90% [1,2], and in the Netherlands PM is chosen by 35–51% of healthy women with a BRCA1/2 mutation [3,4].

Since the decision to pursue PM is complex, personal, and ultimately irreversible, it has been suggested that decision-making should include counseling of the woman by a multidisciplinary team of specialists, addressing the benefits of the different procedures as well as the surgical and psychological risks of PM [5]. The information perceived by women as being most important with respect to the decision for risk-reductive surgery was a BRCA test result and discussion of the family cancer history, whereas reasons for persisting indecision about surgery included genetic test results, concerns about surgery as a procedure, and timing in life [6].

Women who elected to undergo PM reported experiencing higher levels of anxiety associated with developing breast cancer than those women who did not opt for surgery [7]. Studies have shown that the majority of women following PM reported decreased (hereditary) cancer related distress while other psychosocial functioning does not appear to be significantly affected in a negative way [5,8-10]. Post-surgical distress appeared to be related to surgical complications, perceived risk of breast cancer, having young children, and psychiatric history [11-14].

Such factors as they relate to distress in women warrant additional attention, and several authors have recommended to (further) identify women at psychological risk in order to provide the appropriate psychological or psychiatric care [14,15]. While there are high levels of endorsement for the provision of psychological consultation before and after PM by women considering surgery, and pre-surgical psychological consultation is available in some centers, it is by no means a routine, integrated component of the pre- or post-surgical care of women who undergo PM in most settings [16].

As of the start of the Rotterdam Family Cancer Clinic in 1991 prophylactic surgery was discussed with either healthy women (unaffected) or women with a history of breast or ovarian cancer (affected) after exclusion of recurrent disease by standard dissemination examinations. The counseling sessions, the surveillance programs and/or (preventive) interventions for BRCA1/2 mutation carriers and for women at increased risk of hereditary breast/ovarian cancer, were carried out by a multidisciplinary team of medical, surgical, and gynecological oncologists, in collaboration with clinical geneticists. Apart from separate information sessions for the high-risk women with the different specialists of the family cancer clinic team, each case was discussed separately and consensus regarding policy was sought at the scheduled multidisciplinary meetings. This approach was formalized in a written protocol, and approved by the working party on hereditary tumors of our institution. Institutional guidelines became available as of 1995, and were regularly updated.

Because optimal counseling in the process of decision-making regarding PM was considered important and few data were available at that time, the committee on hereditary tumors at our institution decided that, as of 1999, referral to a psychologist would be standard procedure before PM. Aim of this study was to asses: 1) the acceptance of the offer of a standard psychological consultation as part of pre-surgical decision-making in high-risk women, 2) reasons for PM and reasons for postponing it, 3) the need for additional psychological interventions, and factors associated, and 4) the frequency of psychiatric/psychological treatment history in these high risk women.

## Methods and participants

### Psychosocial Oncology Service

The psychologist is a member of the Department of Psychosocial Oncology, a multidisciplinary liaison Psychosocial Oncology Care Service since 1987, where 2 psychiatrists, 1 psychologist, 1 psychiatric nurse specialist, 5 social workers, and 2 chaplains, work in close collaboration with the medical specialists and nursing staff in the management of cancer patients. Psychiatric medications, psychological interventions, advice, spiritual counseling and educational support can be tailored to address specific needs of cancer patients and their families. In providing a standard psychological consultation pre-surgery we hoped to better understand the psychological issues faced by high risk women considering PM during decision making and their need for (additional) psychological support.

### Study population

Between June 1999 and December 2001, 73 women at risk of developing hereditary breast cancer who were considering PM, were routinely referred to the psychologist (M.T.). Criteria for referral to the psychologist were: a high risk of developing breast cancer (either being a carrier of a BRCA1/2 mutation, or being at 50% risk for being a carrier, with or without a history of breast/ovarian cancer, and without disease recurrence), and interest in PM as an option to reduce the breast cancer risk. Partners were also actively encouraged to attend the psychological consultation.

### Psychological consultation

The consultation was performed in a standardized manner. Beforehand, information on demographics and medical history were obtained from the hospital medical record, and the consultation started with verifying this information. Questions were posed about the woman's reaction to the genetic testing result, and the impact of this result for herself, her partner and her family. The women were also asked to discuss their personal experience with cancer (or within their family) and their associated fears. Then, the woman's motivations and reasons to undergo PM, and the assumed consequences of the PM regarding sexuality, were addressed by means of the following questions: *"Can you tell me why you are considering prophylactic mastectomy at this moment? Have you (both) spoken about the consequences of the surgery for your sexual life and, if so, can you tell me about that?" *Moreover, we asked about any issues concerning the hospital admission, and their support system, using the following question: *"Is there anything we have to do, or can do, to support you during your stay in the hospital?" *Finally, we asked if there was any personal history of psychological/psychiatric treatment and, if so, what were the reason(s) for this, and how was this treated.

At the end of the consultation (lasting about one hour), according to the expert rating, the psychologist and the counselee decided whether psychological support was needed or was considered helpful. Additional psychological help was supportive orientated and existed of individual psychological interventions and counseling for the women and their partners. In case of (continuing) need for psychotropic medication, antidepressant medication, this was administered by the liaison psychiatrist. Psychological support started either after the consultation, during, and/or after the hospital admission for the PM, and was always proposed actively in case of current mental problems and/or burdensome psychosocial circumstances. In case of relevant mental problems, such as mood disorders and anxiety disorders (based on DSM-IV criteria [17]), the PM was postponed. A summary of the psychological consultation was made and sent to the physicians involved and also, on request, to the counselees. Specific concerns, i.e. present mental illness, ongoing uncertainty about the decision to undergo PM, or when extra decisional counseling sessions were considered necessary, were communicated by the psychologist at the scheduled multidisciplinary meetings.

### Data collection and statistical analysis

Data abstracted from the medical file included: age, genetic risk status, cancer status, cancer site, and family cancer history. Using a self-developed checklist, the following variables were obtained from the psychological consultation: primary reasons for opting or declining surgery, current psychological distress, need for additional psychological support, and previous psychological/psychiatric treatment. In cases of new psychological consultations over time reasons for referral and relevant problems as stated by the referring doctor were collected.

Differences between women with and without additional psychological support, respectively, were tested by a t-test (for continuous variables) or a Chi-square test (for categorical variables). A p-value less than 0.05 was considered statistically significant. All analyses were performed with STATA SE software (version 9).

## Results

### Participants

In total, 70 of the 73 women (96%) who were routinely referred to the psychologist before PM were interviewed. Three women declined the psychological consultation, despite receiving a second invitation by letter; two of these three women proceeded with the surgery. The median period between the consultation and the follow-up for this analysis was 26 (range 24–50) months. Of the 70 interviewed women, 57 (81%) proceeded with PM, while 13 (19%) reconsidered or postponed the decision with respect to PM within the range of the follow-up period.

Additional file 1, Table S1 presents the characteristics of the total group of 70 interviewed women. The study sample consisted mainly of unaffected women, and BRCA1/2 mutation carriers (n = 52; 74%). Unaffected women considering PM were significantly younger than affected women (38 vs 48 years; p = 0.002). Of the 18 women without a confirmed mutation, 8 (44%) had been treated for breast cancer (See Additional file 1, Table S1).

### Motivating factors for proceeding with or declining PM

The motivating factors mentioned by the women opting for PM are shown in Additional file 1, Table S2. Reduction of the anxiety for, and the risk of cancer; attempting to break the vicious circle of developing cancer and/or dying from cancer in the family; and feeling responsible for family, spouse and children, were the three most prominent reasons for PM. The reasons for declining or postponing PM were known for all 13 women (Additional file 1, Table S3) and mostly included: uncertainty to proceed with surgery because they needed more time and information to make a final decision, and the need for a better understanding of the risk estimation (See Additional file 1, Table S2 and Table S3).

### Extra psychological support

**Figure 1:**
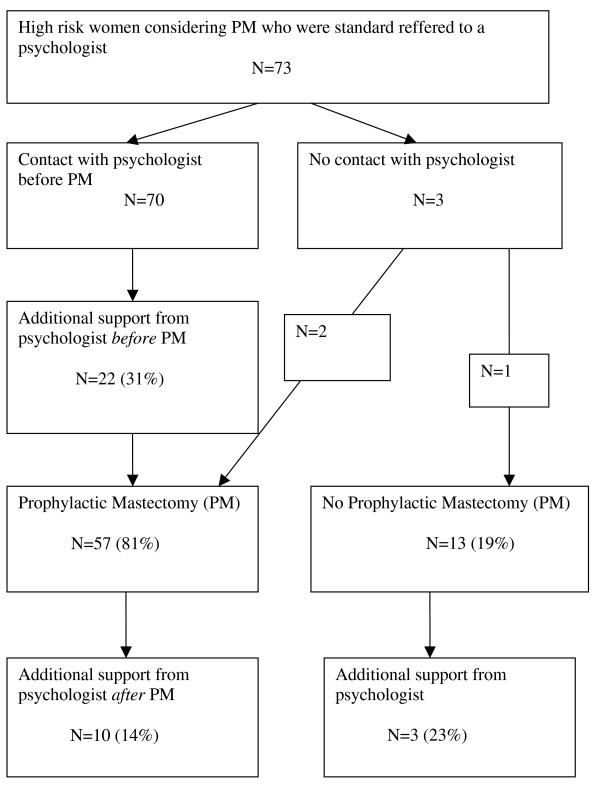


Of the interviewed women, 22 (31%) received additional psychological help by the same psychologist who interviewed them. Six women (27%) had 1–2 extra counseling sessions before the hospital admission for PM to help them cope with the upcoming surgery. Fourteen women (64%) were visited 1–3 times during their admission in the hospital for PM. Ten women (14%) received additional psychological support after PM. Major depression was diagnosed in 3 women (30%) following PM and all received antidepressant medication, 1 woman had her first major depressive episode, 2 women had a recurrence of depression at this time. Four women received psychological support for adjustment problems after surgery (40%), and 3 women (30%) received further bereavement counseling. Eventually, 4 of these 10 (40%) women were referred to an outpatient Mental Health provision for further support. Figure 1 shows a flow chart of support over time (see Figure 1).

Of the 13 women who reconsidered or postponed their decision for PM, 3 (23%) received extra psychological support with a total amount of 19 interventions within 1 year. One woman was too anxious to come to the hospital and unable to go through with the prophylactic surgery. Another woman (who was treated for breast cancer 1.5 years before considering PM) realized that she needed extra information with respect to risk estimation. Finally, insurance problems with respect to breast reconstruction emerged for one woman who later decided to undergo breast reconstruction in another country.

Additional file 1, Table S4 presents the characteristics of the women who were (n = 22), or were not receiving additional psychological support (n = 48). Of the 22 women receiving extra support, 10 (45%) had a history of psychiatric/psychological treatment, versus 15 of the 48 women (31%) not requiring additional psychological support (p = 0.25). The uptake for additional psychological support was significantly higher in women with a proven BRCA1 or BRCA2 mutation (91% vs 67%; p = 0.03), and tended to be higher in women with experience of death due to cancer of a sister (23% vs 2%; p = 0.07). Of the 57 women who proceeded with PM, 19 (33%) received extra psychological support. Cancer status and whether or not proceeding with PM did not differ between women receiving or not receiving extra psychological support (see Additional file 1, Table S4).

### Reported history of psychiatric/psychological treatment

Of the interviewed women, 25 (36%) reported a history of psychiatric/psychological treatment and had all been treated by health professionals at an out-patient Mental Health provision in The Netherlands. Thirteen women (52%) were treated for affective disorders, (recurrent) depressions, for which they received antidepressant medication psychotherapy. Six women (24%) had been treated for stress related disorders: 1 after a suicide attempt, 2 after a divorce, 2 after burn out, and 1 relating to the diagnosis of cancer. Six women (24%) were referred for psychotherapeutic interventions by their GP because of bereavement due to death of a mother.

## Discussion and conclusion

### Discussion

This long-term study assessed the acceptance of the offer of a psychological consultation as standard practice pre-surgical, primary reasons in (genetically) high-risk women considering (not) undergoing prophylactic mastectomy, the need for additional support, and reported data on a psychiatric/psychological treatment history. Our study sample was relatively large, including 70 high-risk women from a single-center family cancer clinic. The most important findings of our analysis are: 1) 70 out of 73 women accepted the psychological consultation as standard practice pre-surgery; 2) main reasons to undergo PM were to reduce anxiety for and the risk of developing cancer, and to avoid repetition of the family cancer history, 19% of the interviewed women reconsidered or postponed the PM because of 'uncertainty about the decision' and 'a need for more risk information'; 3) 31% of the women received additional psychological support; and 4) 36% of the women considering PM reported a history of psychiatric/psychological treatment.

In the present study, the two main reasons for women to opt for PM were: to reduce anxiety for and the risk of developing cancer, and to avoid a repetition of the family cancer history. The two most important reasons to decline or postpone PM were: uncertainty to proceed with surgery, and the need for a better understanding of the risk estimation. These results are consistent with the findings of others [9,18,19], and indicate that the information provided at the family cancer clinic is complex, thus stressing the importance of repetitive but consistent information regarding the cancer risk by the different clinicians involved. Referral to the psychologist is relevant in this respect to help explore the quality of decision-making, and to determine which additional information is needed to help women make an optimal informed decision.

In our study sample, 1 in 3 women opting for and proceeding with PM received extra psychological support; 10 women continued to receive additional psychological support after surgery, and 3 of them required antidepressants. The latter finding is in accordance with Hopwood *et al*. [20], who reported that 16% (7/45) of their group required further psychiatric help following PM, and antidepressant medication was used by 3 women. In our study sample, the most important factors associated with additional psychological support were genetic risk status, and the experience with cancer in a close relative. Although, our small study subgroups do not allow more detailed statistical analyses, the latter findings are supported by Erblich *et al*. [21] who found the highest distress scores among women whose mother had died from breast cancer and who had been involved in the care of their mother during the cancer process. Similarly, Van Dooren *et al*. [22] found that having a sister with breast cancer and being involved in her disease process was more distressing to women than having experienced the process of breast cancer in a mother. Thus, focusing on this issue in the care of, and in future studies on, high-risk women, is warranted.

The high frequency (36%) of history of psychiatric/psychological treatment (mainly for depressions and bereavement) in high-risk women opting for PM might reflect the emotional distress associated with the process of sickness and death due to cancer in family relatives. This is in accordance with Wellish *et al*. who reported that psychological distress was more pronounced in daughters with a mother affected with breast cancer during adolescence as compared to during adulthood [23]. Similarly, Hopwood *et al*. found that women bereaved of their mothers due to cancer during adolescence had higher cancer worries than women who had lost their mothers during adulthood [24].

To our knowledge, no other data on the rate of psychiatric/psychological treatment history in women opting for PM are available with which to compare our results. According to the National Mental Health Survey and Incidence (NEMESIS) the lifetime observed prevalence rate for psychiatric disorder for women in the Netherlands is 40% [25]. However, as this is a lifetime prevalence in the Dutch female population aged 18 to 64 years, this is not directly comparable with our population. The high rates of psychiatric/psychological treatment history in women opting for PM warrant further research in larger groups, and such research should also include high-risk women opting for intensive surveillance.

### Limitations

Between June 1999 and December 2001, 87 women at increased risk of hereditary breast cancer underwent PM at our clinic. Of these 71% (n = 59) were referred for a psychological consultation. It is most plausible that both logistical problems at the start of this study (the fact that it needed some time to get all the medical specialists in line to routinely refer these women pre-surgery to the psychologist) and patient characteristics (e.g., more vulnerable women were referred) have played a role in the suboptimal referral rate. The observation that the number of unaffected women significantly exceeded that of the affected women, whereas the number of women having undergone PM at our institution consists of about 50% affected women [26], supports the idea that selection bias for referral to the psychologist has indeed occurred. It is therefore unclear to what extent our results may be an overestimation of the percentage in need for additional psychological interventions. Another limitation is the lack of a comparison group of mutation carriers who were not considering PM.

### Conclusion

Our results indicate that genetically susceptible women considering PM are vulnerable, especially mutation carriers and those having been involved in the cancer process and death of a close relative. This should be explored more extensively in future, prospective, studies in order to optimize psychological support. Meanwhile, a standard consultation with the psychologist for high-risk women considering a PM is indicated, in order to help them come to an informed decision, to detect and manage psychological distress, and to plan psychological support services accordingly.

## Competing interests

The authors declare that they have no competing interests.

## Authors' contributions

MT was responsible for the conception of the study, data collection, and the writing of the article. EMAB was responsible for the writing of the article. ARVG and SVD revised the article critically for important intellectual content. MBEM, BNVG, MMAT, KCMB, JGMK contributed to the conception of the study. CTMB was responsible for the analysis and interpretation of the data. CS contributed to the conception of the study and revised the article critically for important intellectual content. All authors have read and approved of the final article.

## Supplementary Material

Additional file 1 **Tables S1, Table S2, Table S3 and Table S4.** Tables. Click here for file
