# Supplementary material for: Standard psychological consultations and follow up for women at increased risk of hereditary breast cancer considering prophylactic mastectomy
Source: Hered Cancer Clin Pract. 2009 Mar 31;7(1):6. doi: 10.1186/1897-4287-7-6 (PMC2667399; doi:10.1186/1897-4287-7-6)
Supplement: Additional file 1 — Tables S1, Table S2, Table S3 and Table S4. Tables. [file 1897-4287-7-6-S1.doc]

Table S1. Characteristics of the interviewed women (n=70)

| *Age (in years):* |  |  |  |
| --- | --- | --- | --- |
| Mean age | 41 |  |  |
|  |  |
| Range | 22-60 |  |  |
|  |  |
| *Genetic risk status: N (%)* | | |  |
| Mutation carrier |  |  |  |
|  |  |
| BRCA 1 | 47 (67%) |  |  |
|  |  |
| BRCA 2 | 5 (7%) |  |  |
|  |  |
| 50% risk carrier from: | | | |
| HBOC family | 4 (6%) |  |  |
|  |  |
| HBC family | 14 (20%) |  |  |
|  |  |
| *Cancer status:* | | |  |
| Unaffected women | 52 (74%) |  |  |
| Mean age (range) | 38 (22-59) |  |  |
|  |  |
| Affected women | 18 (26%) |  |  |
|  |  |
| Mean age (range) | 48 (34-60) |  |  |
|  |  |
| *Type of cancer:* | | | |
| Invasive breast cancer | 13 (72%) |  |  |
|  |  |
| DCIS | 2 (11%) |  |  |
|  |  |
| Ovarian cancer | 3 (17%) |  |  |
|  |  |
| *Psychiatric/psychological treatment history:* | 25 (36%) |  |  |
|  |  |  |  |
| Affective disorders: | |  |  |
| Depressive episode  (recurrent) | 13 (52%) |  |  |
|  |  |  |  |
| Stress related disorders:  Adjustment disorders | 6 (24%) |  |  |
| Psychotherapy with | 6 (24%) |  |  |
| focus on death of mother |  |  |  |

Table 2. Main reasons for proceeding with PM (n=57); more than one reason could be given.

| Reasons | *N (%)* |
| --- | --- |
| Reducing anxiety about cancer as well as reducing the risk itself | 32 (56%) |
| To avoid a repetition of the family cancer history | 26 (46%) |
| Feeling responsible for family, spouse and children | 18 (32%) |
| Lack of trust in detection method and treatment outcome | 13 (23%) |
| To be proactive in trying to prevent cancer | 10 (17%) |

Table 3. Main reasons for declining prophylactic mastectomy (n=13); more than one reason could be given.

| *Reasons* | *N (%)* |
| --- | --- |
| Uncertainty about the decision | 3 (23%) |
| Needing further risk information | 3 (23%) |
| Family planning/pregnancy | 2 (15%) |
| Too young | 2 (5%) |
| Present mental problems | 2 (15%) |
| Wanting to await result of gene testing | 2 (15%) |
| Problems with insurance | 1 (8%) |

Table 4. Characteristics of women receiving additional (n=22), or no additional psychological support (n=48).

|  | Additional support  (n=22)  n ( %) | No additional support (n=48)  n (%) | p-value |
| --- | --- | --- | --- |
| *Age (in years)* |  |  | 0.89 |
| Mean age | 40 | 41 |  |
| Range | 24-60 | 22-59 |  |
| *Psychological treatment history* | 10 (45%) | 15 (31%) | 0.25 |
| *Cancer status:* |  |  | 0.33 |
| Unaffected | 18 (82%) | 34 (71%) |  |
| Affected | 4 | 14 |  |
| Genetic risk status: |  |  | 0.03 |
| Mutation carrier: |  |  |  |
| BRCA1/2 | 20 (91%) | 32 (67%) |  |
| 50% risk carrier from |  |  |  |
| HB(O)C | 2 (9%) | 16 (33%) |  |
| *Surgery status:* |  |  | 0.43 |
| Prophylactic mastectomy | 19 (86%) | 38 (79%) |  |
| PM postponed | 3 (14%) | 10 (21%) |  |
| *Family history:* |  |  | 0.07 |
| Mother died of cancer | 8 (47%) | 25 (60%) |  |
| Mother affected | 2 (12%) | 8 (19%) |  |
| Sister died of cancer | 4 (23%) | 1 (2%) |  |
| Sister affected | 3 (18%) | 8 (19%) |  |
